# Influence of socio-economic status on Shiga toxin-producing *Escherichia coli* (STEC) infection incidence, risk factors and clinical features

**DOI:** 10.1017/S0950268819000864

**Published:** 2019-06-13

**Authors:** N. L. Adams, L. Byrne, T. C. Rose, G. K. Adak, C. Jenkins, A. Charlett, M. Violato, S.J. O'Brien, M. M. Whitehead, B. Barr, D. C. Taylor-Robinson, J. I. Hawker

**Affiliations:** 1 Tuberculosis; Acute Respiratory, Gastrointestinal, Emerging/Zoonotic Infections; and Travel and Migrant Health Division, National Infection Service, Public Health England, London, UK; 2 NIHR Health Protection Research Unit in Gastrointestinal Infections, Liverpool, UK; 3 Department of Public Health and Policy, University of Liverpool, Liverpool, UK; 4 Statistics, Modelling and Economics Division, Public Health England, London, UK; 5 Health Economics Research Centre, University of Oxford, Oxford, UK; 6 Field Service, National Infection Service, Public Health England, Birmingham, UK

**Keywords:** Gastrointestinal infections, health inequalities, Shiga-like toxin-producing *E. coli*

## Abstract

Shiga toxin-producing *Escherichia coli* (STEC) infection can cause serious illness including haemolytic uraemic syndrome. The role of socio-economic status (SES) in differential clinical presentation and exposure to potential risk factors amongst STEC cases has not previously been reported in England. We conducted an observational study using a dataset of all STEC cases identified in England, 2010–2015. Odds ratios for clinical characteristics of cases and foodborne, waterborne and environmental risk factors were estimated using logistic regression, stratified by SES, adjusting for baseline demographic factors. Incidence was higher in the highest SES group compared to the lowest (RR 1.54, 95% CI 1.19–2.00). Odds of Accident and Emergency attendance (OR 1.35, 95% CI 1.10–1.75) and hospitalisation (OR 1.71, 95% CI 1.36–2.15) because of illness were higher in the most disadvantaged compared to the least, suggesting potential lower ascertainment of milder cases or delayed care-seeking behaviour in disadvantaged groups. Advantaged individuals were significantly more likely to report salad/fruit/vegetable/herb consumption (OR 1.59, 95% CI 1.16–2.17), non-UK or UK travel (OR 1.76, 95% CI 1.40–2.27; OR 1.85, 95% CI 1.35–2.56) and environmental exposures (walking in a paddock, OR 1.82, 95% CI 1.22–2.70; soil contact, OR 1.52, 95% CI 2.13–1.09) suggesting other unmeasured risks, such as person-to-person transmission, could be more important in the most disadvantaged group.

## Introduction

Shiga toxin-producing *Escherichia coli* (STEC, also known as verocytotoxin-producing *E. coli* or VTEC) are a group of bacteria that cause infectious gastroenteritis, with STEC serogroup O157 being the most frequently reported strain causing illness in England. Transmission to humans occurs through consumption of contaminated food or water, exposure to a contaminated environment involving direct or indirect contact with animals or their faeces. The low infectious dose of STEC means that once in a population, person-to-person spread is common [1–3].

Infection with STEC is a relatively rare cause of gastrointestinal illness in England, with around 900 cases reported annually. However, symptoms can range from mild gastroenteritis through to severe bloody diarrhoea and infection can cause the serious condition of haemolytic uraemic syndrome (HUS), affecting the blood, kidneys and, in the most severe cases, the central nervous system. Children and the elderly are most susceptible to severe illness and HUS is recognised as the most common cause of acute renal failure amongst children in the UK [4]. Despite many interventions to reduce the incidence of STEC infection over the last 30 years, which have resulted in changes in risk factors, levels of infection have remained relatively stable [3].

Risk factors for STEC infection are well documented and include a variety of foodborne, waterborne and environmental factors as well as non-UK travel [5–7]. The relationship between STEC infection, risk factors and socio-economic status (SES) is less clear [8]: two studies from the USA and one from Finland [9–11] reported that low SES is associated with a lower risk of STEC infection and potentially a lower risk of progression to HUS [10, 12]. However, a study from Japan [13] reported the opposite pattern and studies from Canada [14] and Denmark [15] found no clear association with SES. A recent systematic review focusing on the relationship between SES and gastrointestinal infections in developed countries did not identify any high-quality studies relating to STEC [16]. Studies reporting differences in the risk of GI infection by SES have hypothesised that these may be due to differential exposure to non-UK travel, eating outside of the home or dietary risk factors [9–11]; or related to differential healthcare-seeking behaviour [10]; however, to the best of our knowledge, there are no studies exploring the social patterning of risk factors for STEC or clinical presentation. This study therefore aims to investigate, within a population of STEC cases, whether there are differences by SES in (i) STEC incidence, (ii) clinical presentation (symptoms and healthcare usage) and (iii) STEC risk factors, using the National Enhanced Surveillance System for STEC (NESSS), to assess the direction of any associations identified and to suggest hypotheses for testing in future studies, which could inform interventions to reduce STEC infection.

## Methods

### Data, setting and source

An observational study design was used to assess the relationship between SES and a variety of clinical and risk factors for STEC infection amongst STEC cases utilising data extracted from the Public Health England (PHE) NESSS, described in detail elsewhere [17]. Results from the microbiological characterisation of STEC cases are included in NESSS, alongside standardised information collected through administration of an enhanced surveillance questionnaire (ESQ). The ESQ collects detailed information on patient demographics, symptoms, food and water risk factors and the UK and non-UK travel during the exposure period (the week prior to illness onset). Risk factor questions included in the ESQ reflect evidence-based knowledge and refer to known risk factors documented in the literature as well as risk factors identified as part of outbreak investigations [1, 5–7, 18–21], for example, relating to petting farm visits or contaminated meat products. We extracted demographic, clinical, microbiological and risk factor data collected on all symptomatic STEC cases with an ESQ reported to NESSS between 1 January 2010 and 31 December 2015 (inclusive). Exposure data were available for all STEC cases included in this study. We included sporadic and outbreak-associated cases.

SES was determined using a small-area deprivation measure, the Index of Multiple Deprivation 2010 (IMD) [22], assigned to each individual based on their full postcode and categorised into population-level quintiles. Where symptoms, travel status, healthcare contact or risk factor variables were blank or unknown, these were recoded as a negative response.

### Ethics

This study falls under the existing Health Protection Agency (HPA, now PHE) permissions under Section 251 of the NHS Act 2006. In addition, a favourable ethical opinion was received from the South East Coast – Surrey Research Ethics Committee (15/LO/2138) on 1 December 2015 covering the use of this dataset.

### Outcome and exposures

The primary exposure of interest was SES. The outcomes of interest were a range of reported foodborne, waterborne and environmental risk factors as well as clinical presentation which were coded as binary variables (yes/no). The association between each reported risk factor and the primary exposure of interest (SES) was tested.

### Descriptive methods

Analyses were conducted in Stata 13.1 (Statacorp, Texas, USA).

To take account of the underlying population ‘at risk’, crude STEC rates per 100 000 population were calculated for each IMD quintile. Age- (0–4, 5–9, 10–15, 16–19, 20–59 and 60+) and sex-specific incidence rates were also calculated for each IMD quintile.

A descriptive analysis of the distribution of case characteristics, clinical factors, healthcare contact and risk factors by IMD quintiles was undertaken. A *χ*^2^ test for trend was used to assess the relationship between IMD quintile and each of the case characteristics in turn. All case characteristic variables were retained for inclusion *a priori* in the subsequent analyses, with the exception of the UK and non-UK travel and outbreak association.

For the clinical factors, healthcare contact and risk factors, a *χ*^2^ test was used to assess whether there was a statistically significant relationship between IMD quintile and each of the variables in turn in order to select variables for inclusion in further analysis. The categories for these variables were not mutually exclusive as individuals could report exposure to multiple factors. Variables identified as significantly related to IMD in this descriptive analysis (*P* ⩽ 0.05) were included in subsequent analyses.

### Analytical methods

Due to missing data (19.1%) for the ethnicity variable (White/non-White), multiple imputation using chained equations [23] was used to impute missing values in order to include ethnicity as a variable in the models. Fifty imputed datasets were generated. The distribution of ethnicity by age and sex was assessed to check the missing at random assumption.

Univariate and multivariable logistic regression models were used to assess the relationship between IMD quintile and clinical features. Age, sex, ethnicity, rurality and *stx* gene, associated with increased severity, were included *a priori* in the multivariable analysis.

The relationship between IMD and non-UK travel was assessed using univariate and multivariable logistic regression. Non-UK travel-associated cases were then excluded from the analysis, as risk factor data are not routinely collected from these cases, and univariate and multivariable logistic regression was used to assess the relationship between IMD and each potential risk factor variable (food, water, environmental and UK-travel risk factors) in turn. Age, sex, ethnicity and rurality were included *a priori* in the multivariable analysis.

### Robustness tests

To assess the robustness of our findings, the risk factor analysis was repeated for sporadic cases and for cases aged <16 years to determine whether there were differences in risk factors by SES for children. The clinical presentation analyses were repeated on restricted datasets for (i) non-travel-associated cases, (ii) sporadic cases and (iii) children aged <16 years.

## Results

### Descriptive results

A total of 4200 primary, symptomatic STEC cases were reported to NESSS between 1 January 2010 and 31 December 2015, 98% of which (*n* = 4115) had an ESQ. It was not possible to match the postcode to an IMD score for 143 cases (3.47%). Therefore, 3972 cases of STEC were included in our study. Non-UK travel was reported by 1011 STEC cases (25.5%). Information on exposure to risk factors was available for all 2961 non-travel cases. Ethnicity data were available for 80.9% of cases and imputed for the remainder of the main analysis (see Table 1 for STEC distribution by ethnicity). There was no difference in missing ethnicity by sex or age group.

**Table 1.** Characteristics of STEC cases by Index of Multiple Deprivation quintile (*n* = 3972)

	Q1 (least disadvantaged)	Q2	Q3	Q4	Q5 (most disadvantaged)	*P* value^a^
	*n* (%)	*n* (%)	*n* (%)	*n* (%)	*n* (%)	
Total	945 (23.8)	913 (23.0)	821 (20.7)	699 (17.6)	594 (15.0)	<0.01^b^
Age group						
<1	14 (19.2)	18 (24.7)	9 (12.3)	13 (17.8)	19 (26.0)	
1–4	133 (19.8)	142 (21.1)	154 (22.9)	125 (18.6)	118 (17.6)	
5–9	97 (24.4)	95 (23.9)	83 (20.9)	60 (15.1)	63 (15.8)	
10–15	99 (29.1)	74 (21.8)	67 (19.7)	55 (16.2)	45 (13.2)	
16–19	69 (30.8)	57 (25.4)	38 (17.0)	33 (14.7)	27 (12.1)	
20–59	357 (22.8)	353 (22.6)	321 (20.5)	294 (18.8)	239 (15.3)	
60+	176 (25.1)	174 (24.8)	149 (21.3)	119 (17.0)	83 (11.8)	
Sex						
Male	412 (24.1)	381 (22.3)	345 (20.2)	288 (16.8)	284 (16.6)	
Female	533 (23.6)	532 (23.5)	476 (21.0)	411 (18.2)	310 (13.7)	0.29
Ethnicity						
White	758 (26.0)	706 (24.2)	634 (21.8)	475 (16.3)	341 (11.7)	
Non-White	28 (9.4)	32 (10.7)	34 (11.4)	73 (24.4)	132 (44.1)	
Unknown	159 (20.9)	175 (23.1)	153 (20.2)	151 (19.9)	121 (15.9)	
Rurality						
Urban	671 (23.0)	542 (18.6)	512 (17.6)	605 (20.8)	582 (20.0)	
Rural	274 (25.8)	371 (35.0)	309 (29.2)	94 (8.9)	12 (1.1)	<0.001
*Stx*						
*Stx*1 + 2	385 (26.0)	354 (23.9)	306 (20.7)	249 (16.8)	186 (12.6)	
*Stx*1	35 (25.9)	26 (19.3)	20 (14.8)	27 (20.0)	27 (20.0)	
*Stx*2	525 (22.3)	533 (22.6)	495 (21.0)	423 (17.9)	381 (16.2)	
UK travel	203 (26.2)	198 (25.5)	163 (21.0)	139 (17.9)	73 (9.4)	<0.001
Non-UK travel	293 (29.0)	220 (21.8)	180 (17.8)	175 (17.3)	143 (14.1)	0.003
Outbreak-associated	154 (26.3)	145 (24.8)	131 (22.4)	83 (14.2)	72 (12.3)	0.003

a Statistical significance of the relationship between IMD quintile and each variable, tested using *χ*^2^ test for trend.

b Statistical significance of relationship tested using *χ*^2^ test.

**Fig. 1.**
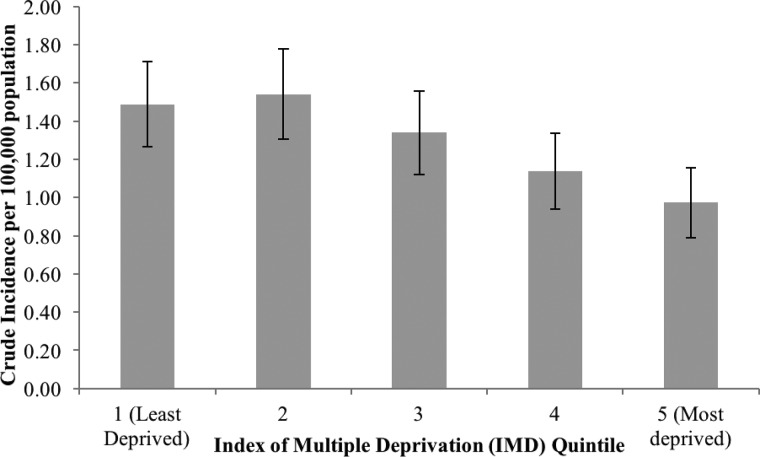
Crude incidence of STEC infection (per 100 000 population) by socio-economic status.

The overall incidence of STEC for England was 1.30 per 100 000 population (95% CI 1.20–1.39). Incidence was highest in the 0–4 age group at 3.90 per 100 000 population (95% 3.21–4.58), statistically significantly higher than any other age group. The crude rate in females (1.45/100 000, 95% CI 1.30–1.60/100 000) was statistically significantly higher than in males (1.14/100 000, 95% CI 1.00–1.27/100 000, Table 2). Crude incidence rates increased as deprivation decreased with incidence in the least disadvantaged significantly higher than the incidence in the most disadvantaged (IRR 0.65, 95% CI 0.50–0.84; Fig. 1). The pattern of higher rates in the least *vs.* the most deprived quintile was also reflected in the age- and sex-specific incidence rates for older age groups (from age 10), but children aged under 10 showed the opposite pattern, although the observed IRRs were not statistically significant for any age group except for those aged 60 years and over. (Table 2).

**Table 2.** Incidence rate ratio for exposed (most disadvantaged) compared to unexposed (least disadvantaged)

	Crude rate/100 000 (95% CI)	Rate/100 000 in most disadvantaged	Rate/100 000 in least disadvantaged	Incidence rate difference	Incidence rate ratio	95% CI
Overall	1.30 (1.20–1.39)	0.97	1.49	−0.51	0.65	0.50–0.84
Female	1.45 (1.30–1.60)	1.00	1.65	−0.66	0.61	0.42–0.86
Male	1.14 (1.00–1.27)	0.95	1.32	−0.37	0.71	0.48–1.06
0–4	3.90 (3.21–4.58)	3.84	3.25	0.59	1.17	0.63–2.14
5–9	2.31 (1.76–2.87)	1.89	2.57	−0.68	0.78	0.33–1.79
10–15	1.57 (1.16–1.97)	1.04	2.25	−1.21	0.48	0.18–1.17
16–19	1.45 (0.99–1.92)	0.89	2.27	−1.38	0.42	0.12–1.27
20–59	0.96 (0.84–1.07)	0.76	0.96	−0.20	0.79	0.51–1.19
60+	1.01 (0.83–1.19)	0.54	1.65	−1.10	0.34	0.16–0.66

### Analytical results

Bloody diarrhoea, nausea, abdominal pain, fever, HUS, antibiotics, antidiarrhoeals and contact with NHS Direct were not significantly associated with SES in the descriptive analysis (Table 3) and were excluded from subsequent analysis. In multivariable analysis (Table 4) accounting for age, sex, ethnicity, rurality and potential severity (defined by s*tx* gene), those who were more disadvantaged were more likely to report vomiting (OR 1.61, *P* < 0.001). They were also more likely to report visiting A&E or being hospitalised for their illness (OR 1.35, *P* = 0.02; OR 1.71, *P* < 0.001, respectively), but less likely to report visiting their GP (OR 0.67, *P* < 0.01). There was no significant difference by SES for reporting of diarrhoea. There was also no significant difference in odds of being associated with an outbreak by SES.

**Table 3.** Exposures and clinical presentation of STEC cases by Index of Multiple Deprivation quintile

	Q1 (least disadvantaged)	Q2	Q3	Q4	Q5 (most disadvantaged)	*P* value^a^
Clinical factors (*n* = 3972)						
Diarrhoea	917 (97.0)	858 (94.0)	785 (95.6)	669 (95.7)	573 (96.5)	0.02
Bloody diarrhoea	602 (63.7)	572 (62.6)	558 (68.0)	443 (63.4)	386 (65.0)	0.17
Nausea	470 (49.7)	436 (47.8)	403 (49.1)	328 (46.9)	295 (49.7)	0.76
Vomiting	318 (33.7)	344 (37.7)	298 (36.3)	254 (36.3)	259 (43.6)	<0.01
Abdominal pain	815 (86.2)	771 (84.5)	683 (83.2)	580 (83.0)	486 (81.8)	0.15
Fever	316 (33.4)	291 (31.9)	283 (34.5)	246 (35.2)	208 (35.0)	0.60
HUS	28 (3.0)	36 (3.9)	40 (4.9)	29 (4.2)	21 (3.5)	0.33
Healthcare (*n* = 3972)						
Antibiotics	133 (14.1)	128 (14.0)	102 (12.4)	102 (14.6)	77 (13.0)	0.72
Antidiarrhoeals	235 (24.9)	213 (23.3)	172 (21.0)	147 (21.0)	125 (21.0)	0.19
NHS Direct	113 (12.0)	96 (10.5)	87 (10.6)	68 (9.7)	47 (7.9)	0.15
GP	665 (70.4)	637 (69.8)	576 (70.2)	452 (64.7)	367 (61.8)	0.001
A&E	204 (21.6)	195 (21.4)	168 (20.5)	199 (28.5)	174 (29.3)	<0.001
Hospitalised	284 (30.1)	299 (32.8)	286 (34.8)	251 (35.9)	232 (39.1)	<0.01
Food exposures (*n* = 2961)						
Ate outside the home	492 (75.5)	515 (74.3)	458 (71.5)	376 (71.7)	307 (68.1)	0.06
Meat	569 (87.3)	602 (86.9)	552 (86.1)	465 (88.7)	391 (86.7)	0.75
Fish/shellfish	354 (54.3)	325 (46.9)	315 (49.1)	254 (48.5)	200 (44.4)	0.01
Dairy	575 (88.2)	612 (88.3)	572 (89.2)	452 (86.3)	386 (85.6)	0.32
Salad/fruit/vegetables/herbs	540 (82.8)	562 (81.1)	512 (79.9)	413 (78.8)	338 (74.9)	0.02
Juice	267 (41.0)	261 (37.7)	245 (38.2)	191 (36.5)	178 (39.5)	0.56
Water exposures (*n* = 2961)						
Mains water	586 (89.9)	621 (89.6)	553 (86.3)	455 (86.8)	391 (86.7)	0.13
Private water supply	15 (2.3)	35 (5.1)	43 (6.7)	17 (3.2)	1 (0.2)	<0.001
Bottled water	272 (41.7)	224 (32.3)	255 (39.8)	197 (37.6)	175 (38.8)	<0.01
Unboiled water	11 (1.7)	12 (1.7)	12 (1.5)	8 (1.5)	2 (0.4)	0.36
Recreational freshwater exposure	123 (18.9)	129 (18.6)	108 (16.9)	67 (12.8)	49 (10.9)	<0.001
Recreational seawater exposure	37 (5.7)	39 (5.6)	36 (5.6)	28 (5.3)	13 (2.9)	0.22
Environmental exposures (*n* = 2961)						
Any animal contact	447 (68.6)	471 (68.0)	432 (67.4)	340 (64.9)	252 (55.9)	<0.001
Walked in paddock	149 (22.9)	205 (29.6)	175 (27.3)	99 (18.9)	40 (8.9)	<0.001
Visited a farm	91 (14.0)	122 (17.6)	104 (16.2)	62 (11.8)	48 (10.6)	<0.01
Day trip	156 (23.9)	165 (23.8)	150 (23.4)	102 (19.5)	71 (15.7)	<0.01
Contact with soil	168 (25.8)	205 (29.6)	154 (24.0)	104 (19.9)	61 (13.5)	<0.001

HUS, haemolytic uraemic syndrome; s*tx*, shiga toxin type; NHS Direct, National Health Service telephone advice line, now NHS 111; GP, general practitioner; A&E, Accident and Emergency.

a Statistical significance of the relationship between IMD quintile and each variable, tested using *χ*^2^ test.

**Table 4.** Univariate and multivariable regression analysis – clinical factors, healthcare contact and outbreak association (*n* = 3972)

Exposure variable	IMD quintile	Univariate		Multivariable^a^		*P* value
		Odds ratio	(95% CI)	Odds ratio	(95% CI)	
Diarrhoea	1 (least disadvantaged)	1.0 (reference)		1.0 (reference)		
	2	0.48	(0.30–0.76)	0.46	(0.29–0.74)	<0.01
	3	0.67	(0.40–1.10)	0.67	(0.40–1.11)	0.12
	4	0.68	(0.40–1.15)	0.78	(0.46–1.34)	0.37
	5 (most disadvantaged)	0.83	(0.47–1.48)	1.12	(0.61–2.06)	0.72
Vomiting	1 (least disadvantaged)	1.0 (reference)		1.0 (reference)		
	2	1.19	(0.99–1.44)	1.20	(0.99–1.46)	0.06
	3	1.12	(0.92–1.37)	1.13	(0.93–1.38)	0.23
	4	1.13	(0.92–1.38)	1.17	(0.95–1.45)	0.15
	5 (most disadvantaged)	1.52	(1.23–1.88)	1.61	(1.28–2.02)	<0.001
GP	1 (least disadvantaged)	1.0 (reference)		1.0 (reference)		
	2	0.97	(0.80–1.19)	0.95	(0.77–1.16)	0.58
	3	0.99	(0.81–1.21)	0.97	(0.79–1.19)	0.74
	4	0.77	(0.63–0.95)	0.76	(0.62–0.95)	0.01
	5 (most disadvantaged)	0.68	(0.55–0.85)	0.67	(0.53–0.84)	<0.01
A&E	1 (least disadvantaged)	1.0 (reference)		1.0 (reference)		
	2	0.99	(0.79–1.23)	1.05	(0.84–1.32)	0.67
	3	0.93	(0.74–1.18)	0.99	(0.78–1.24)	0.90
	4	1.45	(1.15–1.81)	1.39	(1.10–1.75)	0.01
	5 (most disadvantaged)	1.50	(1.19–1.90)	1.35	(1.05–1.74)	0.02
Hospital	1 (least disadvantaged)	1.0 (reference)		1.0 (reference)		
	2	1.13	(0.93–1.38)	1.15	(0.94–1.40)	0.17
	3	1.24	(1.02–1.52)	1.27	(1.04–1.56)	0.02
	4	1.30	(1.06–1.61)	1.41	(1.14–1.74)	<0.01
	5 (most disadvantaged)	1.49	(1.20–1.85)	1.71	(1.36–2.15)	<0.001
Outbreak	1 (least disadvantaged)	1.0 (reference)		1.0 (reference)		
	2	0.97	(0.76–1.24)	1.03	(0.80–1.32)	0.84
	3	0.98	(0.76–1.26)	1.03	(0.80–1.34)	0.80
	4	0.69	(0.52–0.92)	0.71	(0.53–0.96)	0.02
	5 (most disadvantaged)	0.71	(0.52–0.96)	0.79	(0.58–1.09)	0.16

*Stx*, Shiga toxin gene; GP, general practice; A&E, Accident and Emergency.

a Adjusted for age group, sex, ethnicity, rurality and *stx* gene.

Those who were less disadvantaged were significantly more likely to report non-UK travel (OR 1.76, *P* < 0.001, Table 5).

**Table 5.** Univariate and multivariable regression analysis – non-UK travel (*n* = 3972)

		Univariate		Multivariable^a^		
Exposure variable	IMD quintile	Odds ratio	(95% CI)	Odds ratio	(95% CI)	*P* value
Non-UK travel	1 (least disadvantaged)	1.0 (reference)		1.0 (reference)		
	2	0.71	(0.58–0.87)	0.74	(0.60–0.91)	0.01
	3	0.62	(0.50–0.77)	0.65	(0.52–0.81)	<0.001
	4	0.74	(0.60–0.93)	0.64	(0.51–0.81)	<0.001
	5 (most disadvantaged)	0.71	(0.56–0.89)	0. 57	(0.44–0.73)	<0.001

a Adjusted for age group, sex, ethnicity and rurality.

Eating outside of the home, meat, dairy and drinking juice, mains water, unboiled water and recreational seawater exposures were not significantly associated with SES in the descriptive analysis (Table 3) and were excluded from subsequent analysis. In the multivariable analysis amongst non-travel STEC cases, the least disadvantaged group was statistically significantly more likely to report exposure to fish/shellfish, salad/fruit/vegetables/herbs, private water supplies, recreational freshwater sources, walking in a paddock, taking a day trip, contact with soil or travel within the UK than the least disadvantaged group, after adjusting for age group, sex, ethnicity and rurality (Table 6).

**Table 6.** Univariate and multivariable regression analysis – risk factors (*n* = 2961)

		Univariate		Multivariable^a^		
Exposure variable	IMD quintile	Odds ratio	(95% CI)	Odds ratio	(95% CI)	*P* value
Any fish/shellfish	1 (least disadvantaged)	1.0 (reference)		1.0 (reference)		
	2	0.74	(0.60–0.92)	0.73	(0.59–0.90)	<0.01
	3	0.81	(0.65–1.01)	0.79	(0.64–0.99)	0.04
	4	0.79	(0.63–1.00)	0.78	(0.62–0.99)	0.04
	5 (most disadvantaged)	0.67	(0.53–0.85)	0.67	(0.52–0.87)	<0.01
Any salad/fruit/vegetables/herbs	1 (least disadvantaged)	1.0 (reference)		1.0 (reference)		
	2	0.89	(0.67–1.18)	0.84	(0.64–1.12)	0.24
	3	0.82	(0.62–1.09)	0.76	(0.57–1.02)	0.06
	4	0.77	(0.58–1.03)	0.76	(0.56–1.02)	0.07
	5 (most disadvantaged)	0.62	(0.46–0.82)	0.63	(0.46–0.86)	<0.01
Private water supply	1 (least disadvantaged)	1.0 (reference)				
	2	2.26	(1.22–4.18)			
	3	3.05	(1.68–5.55)	Omitted due to small numbers		
	4	1.42	(0.70–2.88)			
	5 (most disadvantaged)	0.09	(0.01–0.72)			
Bottled water	1 (least disadvantaged)	1.0 (reference)		1.0 (reference)		
	2	0.67	(0.53–0.83)	0.66	(0.53–0.83)	<0.001
	3	0.92	(0.74–1.15)	0.90	(0.72–1.13)	0.36
	4	0.84	(0.67–1.07)	0.78	(0.61–1.00)	0.05
	5 (most disadvantaged)	0.89	(0.69–1.13)	0.80	(0.61–1.04)	0.10
Recreational freshwater exposure	1 (least disadvantaged)	1.0 (reference)		1.0 (reference)		
	2	0.98	(0.75–1.29)	1.02	(0.76–1.37)	0.89
	3	0.87	(0.65–1.16)	0.90	(0.66–1.22)	0.50
	4	0.63	(0.46–0.87)	0.70	(0.49–0.98)	0.04
	5 (most disadvantaged)	0.52	(0.37–0.75)	0.65	(0.44–0.95)	0.03
Recreational seawater exposure	1 (least disadvantaged)	1.0 (reference)		1.0 (reference)		
	2	0.99	(0.62–1.57)	1.03	(0.64–1.65)	0.92
	3	0.99	(0.62–1.59)	1.02	(0.63–1.66)	0.93
	4	0.94	(0.57–1.55)	1.02	(0.61–1.72)	0.93
	5 (most disadvantaged)	0.49	(0.26–0.94)	0.60	(0.31–1.17)	0.13
Any animal contact	1 (least disadvantaged)	1.0 (reference)		1.0 (reference)		
	2	0.97	(0.77–1.22)	0.95	(0.75–1.21)	0.68
	3	0.95	(0.75–1.20)	0.95	(0.74–1.21)	0.67
	4	0.85	(0.66–1.08)	1.09	(0.84–1.42)	0.52
	5 (most disadvantaged)	0.58	(0.45–0.74)	1.01	(0.76–1.33)	0.97
Walked in paddock	1 (least disadvantaged)	1.0 (reference)		1.0 (reference)		
	2	1.42	(1.11–1.81)	1.36	(1.05–1.75)	0.02
	3	1.27	(0.99–1.63)	1.23	(0.94–1.60)	0.13
	4	0.79	(0.59–1.05)	1.04	(0.77–1.40)	0.81
	5 (most disadvantaged)	0.33	(0.23–0.48)	0.55	(0.37–0.82)	<0.01
Visited a farm	1 (least disadvantaged)	1.0 (reference)		1.0 (reference)		
	2	1.32	(0.98–1.77)	1.30	(0.95–1.77)	0.10
	3	1.19	(0.88–1.62)	1.17	(0.85–1.61)	0.34
	4	0.83	(0.59–1.17)	0.90	(0.63–1.29)	0.57
	5 (most disadvantaged)	0.73	(0.51–1.07)	0.94	(0.62–1.40)	0.74
Day trip	1 (least disadvantaged)	1.0 (reference)		1.0 (reference)		
	2	0.99	(0.77–1.28)	1.03	(0.80–1.33)	0.83
	3	0.97	(0.75–1.26)	1.00	(0.77–1.30)	0.99
	4	0.77	(0.58–1.02)	0.79	(0.59–1.06)	0.11
	5 (most disadvantaged)	0.59	(0.44–0.81)	0.64	(0.46–0.89)	0.01
Contact with soil	1 (least disadvantaged)	1.0 (reference)		1.0 (reference)		
	2	0.21	(0.95–1.54)	1.15	(0.90–1.46)	0.28
	3	0.91	(0.71–1.17)	0.88	(0.68–1.14)	0.32
	4	0.71	(0.54–0.94)	0.87	(0.65–1.16)	0.33
	5 (most disadvantaged)	0.45	(0.33–0.62)	0.66	(0.47–0.92)	0.02
UK travel	1 (least disadvantaged)	1.0 (reference)		1.0 (reference)		
	2	0.89	(0.70–1.13)	0.91	(0.71–1.16)	0.44
	3	0.78	(0.60–1.00)	0.80	(0.62–1.03)	0.09
	4	0.80	(0.62–1.05)	0.86	(0.66–1.13)	0.28
	5 (most disadvantaged)	0.46	(0.33–0.62)	0.54	(0.39–0.74)	<0.001

a Adjusted for age group, sex, ethnicity and rurality.

### Robustness tests

Results from restricting the analyses to children aged <16 years differed from the main findings as, unlike for adults, there were no statistically significant differences for contact with a GP, A&E presentation and non-UK travel. However, disadvantaged children were still significantly more likely to be hospitalised. The risk factor analysis for sporadic cases only and clinical presentation for non-travel and sporadic cases did not alter the main findings described above.

## Discussion

In this cross-sectional observational analysis of a dataset of all STEC cases in England reported to NESSS over a 6-year period, the crude incidence of STEC was significantly lower in the most disadvantaged quintile, compared to the least disadvantaged. There were differences in reported exposure to known risk factors by SES; with lower odds of reporting exposure to: salad, fruit, vegetables or herbs; private water supplies; recreational freshwater; walking in a paddock; day trips; contact with soil; UK travel; and non-UK travel amongst the most disadvantaged group. Lower odds of reported exposure to fish/shellfish were also identified. Although this is asked in the ESQ, it is not widely regarded as a risk factor for STEC and likely to reflect incidental differences in population-level exposure by SES. There were also differences in healthcare contact by SES, with lower odds of reporting contact with GP and higher odds of reporting visiting A&E or being hospitalised amongst people living in the most disadvantaged areas.

Two previous studies, in the USA and Finland, found a lower incidence of STEC in communities with lower education levels and hypothesised that this could relate to poorer food safety practices and differential food consumption habits amongst more highly educated individuals [9, 11]. However, the Finnish study also identified an increased incidence of STEC associated with the proportion of low-income households with children [11], indicating that the relationship between SES by age is important for GI infections. The results of our study support the inverse association of STEC incidence with deprivation found in the USA and Finland, rather than positive associations reported in Japan or a lack of association reported in Canada and Denmark [14, 15]. Our study is also consistent with the suggested difference by age reported by the Finnish study (although the positive relationship between incidence and SES in 0–4 year olds in our study was not statistically significant).

In other studies which found higher risk of GI pathogens other than STEC in socio-economically advantaged groups, the authors hypothesised that the finding could relate to: differential exposure, such as consumption of higher risk food amongst those of a higher SES [10, 15, 24–27]; more foreign travel; greater exposure to farm animals or the rural environment. Alternatively, the higher observed risk in high SES groups could be related to differential healthcare-seeking behaviour practices [15, 27]. In our study, there was evidence for differences in non-UK travel, rural environmental exposures and healthcare contact, which would support these assertions. However, there was no evidence to suggest differences in consumption of known foodborne risk factors or greater direct exposure to animals. This may suggest that different risk factors, such as increased opportunities of person-to-person spread, may be more important in disadvantaged groups; a suggestion that has also been postulated by others [8]. This hypothesis warrants further investigation in future studies.

One previous study explored the social patterning of a limited number of potential risk factors for STEC (consumption of hamburgers, type of water available for consumption, recreational water exposure and personal hygiene) in the general population in Argentina [28] as opposed to risk factors reported by cases, as in our study and results are therefore not comparable.

In our study, we observed significantly lower odds of reported non-UK travel amongst disadvantaged STEC cases. It is possible that the pattern of lower odds of reporting non-UK travel amongst more disadvantaged groups reflects the population-level distribution of non-UK travel. Since travel is a known risk factor for STEC infection [5, 17], lower exposure may confer a lower risk of STEC infection.

There is also evidence to suggest that perceptions of attractiveness and safety of local walking environments may differ by SES. Those in more disadvantaged areas may perceive that their local walking environment is less attractive and less safe compared to those in less disadvantaged areas [29]; this could partly explain the lower odds of reporting exposure to walking in a paddock or contact with soil in lower SES groups.

In our study, we also found that the most disadvantaged individuals were less likely to visit their GP but more likely to visit A&E and be hospitalised compared to the least disadvantaged. There is some evidence to suggest that, at the population-level, people living in more disadvantaged circumstances are generally more likely to interact with all three of these healthcare services [30], although this is not specific to GI infections. There is also evidence to suggest that, amongst individuals with GI infections, people living in more disadvantaged circumstances are more likely to be hospitalised [31, 32]. The discrepancy at the GP level could suggest that accessing a GP varies by type of illness for low SES and that there are differences in healthcare-seeking behaviour amongst more disadvantaged individuals with STEC in terms of choosing which services to access; differences in likelihood of milder cases in lower SES group being requested to provide or actually submitting a stool sample (and so not being diagnosed as STEC and appearing in this dataset) or potentially differences in recognition of symptoms at a more advanced stage of illness, delaying seeking of care or differences in access to care. It also raises the possibility that milder cases of STEC infection are less likely to be identified as such in the lower SES group, a trend which has been observed for illnesses in general [30]: this could contribute to our finding of a lower STEC incidence in the lower SES group, which has been identified in other studies reliant on laboratory results for their analysis [33–37]. Finally, it could suggest that although the incidence is lower in the more deprived population, case severity may be higher. However, further studies would be required to robustly assess these hypotheses including a better understanding of why the use of A&E and hospitalisation appear to be higher amongst disadvantaged individuals, despite adjusting for potential severity (*stx* gene). Further work to explore the time between symptom onset and seeking care, as well as differences in stool sampling availability and acceptance, would be of value, as would an exploration of the barriers to seeking care at an earlier stage of illness, to understand whether delayed care is responsible for the higher hospitalisation rate.

A limitation of our study is the cross-sectional observational design. Further research is needed to assess if the associations identified in our study are likely to be causal. Furthermore, the identification of STEC cases included in this study was via laboratory reporting. Laboratory confirmation underestimates the true burden of STEC in a population and such under-reporting may vary by socio-demographic characteristics which may have biased our study population. An area-level measure of SES was used and thus ecological fallacy is a possibility. However, person-to-person spread is an important risk factor for STEC infections and therefore community or area-level risk would be an important factor in considering the individual risk of infection. It has been shown there are larger social networks amongst cases of GI infection in lower socio-economic areas compared to higher socio-economic areas [38]. It was not possible to capture all categories of risk for STEC, including some person-to-person risk factors and more specific food exposures, for example, whether pink or rare meat was served. A further limitation of our study is that no comparable data exist on the specific food and environmental risk factors at the population level by SES. Thus, the differences detected in our study may simply reflect the patterning by SES of these risk factors in the general population. There were also some missing data in our study, particularly for ethnicity, which we addressed using multiple imputation. In addition, since exposure to a private water supply is relatively rare, despite being a known STEC risk factor, it was not possible to perform a multivariable analysis for this due to very small numbers in some covariates. Finally, the lack of significant findings for risk factors in children aged <16 years is likely to be due to low study power since the findings were in the same direction as those in the whole study population.

A strength of our study is the novel analysis of data collected through a country-wide, representative surveillance system to explore the social patterning of risk factors amongst STEC cases in England. Our study thus captures extensive risk factor and exposure data within a well-characterised STEC population. To the best of our knowledge, this is the first study to explore the social patterning of risk factors for STEC in the UK. STEC is a rare but potentially very serious infection and, particularly in children and the elderly, is likely to result in interaction with healthcare and/or public health services. Furthermore, testing of stool samples is routine in UK laboratories. These factors mean it is likely that NESSS captures data on a high proportion of diagnosed STEC cases in England and, hence, this study is likely to be representative of known STEC cases nationally.

In summary, we identified a higher incidence of STEC infection in the highest, compared to the lowest, SES group. There was evidence of social patterning of healthcare contact with more disadvantaged STEC cases having higher odds of A&E presentation and hospitalisation, which may suggest differential healthcare access or consequences of STEC infection by SES. We also found evidence to suggest that some risk factors for STEC are socially patterned, with lower odds of reported exposure to certain known risk factors such as non-UK travel, exposure to salad, fruit, vegetables or herbs and walking in a paddock. This could suggest that certain population groups may be more likely to be exposed to risk factors for STEC, which may partially explain the distribution of STEC cases by SES. Educational programmes geared towards reducing the prevalence of STEC infection should include making higher SES groups aware of their higher risk and specific risk factors. Further research is required to determine the reasons for the differential healthcare contact by SES and particularly for the higher hospitalisation rates observed amongst the lowest SES group. Furthermore, research is needed to determine whether there are risk factors for STEC infection that are more common in lower SES groups.
